# Edge-interior differences in the species richness and abundance of drosophilids in a semideciduous forest fragment

**DOI:** 10.1186/2193-1801-2-114

**Published:** 2013-03-15

**Authors:** Leiza V Penariol, Lilian Madi-Ravazzi

**Affiliations:** Departamento de Biologia, Instituto de Biociências, Letras e Ciências Exatas de São José do Rio Preto/UNESP-IBILCE, Rua Cristovão Colombo, 2265, São Paulo, CEP 15054-000 Brazil

**Keywords:** Forest fragmentation, Drosophilid biodiversity, Bioindicator species, Edge effects

## Abstract

Habitat fragmentation is the main cause of biodiversity loss, as remnant fragments are exposed to negative influences that include edge effects, prevention of migration, declines in effective population sizes, loss of genetic variability and invasion of exotic species. The Drosophilidae (Diptera), especially species of the genus *Drosophila*, which are highly sensitive to environmental variation, have been used as bioindicators. A twelve-month field study was conducted to evaluate the abundance and richness of drosophilids in an edge-interior transect in a fragment of semideciduous forest in São Paulo State, Brazil. One objective of the study was to evaluate the applied methodology with respect to its potential use in future studies addressing the monitoring and conservation of threatened areas. The species abundance along the transect showed a clear gradient, with species associated with disturbed environments, such as *Drosophila simulans*, *Scaptodrosophila latifasciaeformis* and *Zaprionus indianus*, being collected at the fragment edge and the species *D. willistoni* and *D. mediostriata* being found in the fragment’s interior. Replacement of these species occurred at approximately 60 meters from the edge, which may be a reflection of edge effects on species abundance and richness because the species found within the habitat fragment are more sensitive to variations in temperature and humidity than those sampled near the edge. The results support the use of this methodology in studies on environmental impacts.

## Introduction

Fragmentation is a threat to global biodiversity. The fragmentation process affects biodiversity by reducing habitat availability and altering the habitat properties of the remaining fragments (Laurence et al., 2007). Following fragmentation, the primary negative impacts in habitat remnants are edge effects, reduced migration rates, declines in effective population sizes, loss of genetic variability and invasion of exotic species (Fahring, 2003). There are three types of edge effects that influence habitat fragments: (1) abiotic effects, which result from the proximity to a structurally dissimilar matrix and involve changes in environmental conditions; (2) direct biological effects, which involve changes in the abundance and distribution of species, either as a direct result of altered physical conditions or indirectly, as mediated through the physiological tolerances of species to conditions at and near the edge (for example, higher light levels, wind exposure, temperatures and humidity); and (3) indirect biological effects, which involve changes in species interactions, such as predation, brood parasitism, competition and herbivory, biotic pollination and seed dispersal (Murcia, 1995).

According to the Biological Dynamics of Forest Fragments Project (BDFFP), which has evaluated the impacts of fragmentation on the Amazon rainforest and its biota, edge effects are among the most important drivers of ecological change in habitat fragments (Laurance et al., 2011). Today, 32 years after its initiation, BDFFP is the world’s largest and longest-running experimental study of habitat fragmentation as well as one of the most highly cited ecological investigations ever conducted (Gardner et al. 2009; Peres et al. 2010).

Edge size is an important factor in evaluating the environmental impacts within a fragment. Data from the relevant literature indicate that the extent of the edge ranges from 50 to 500 meters (Laurence, 2000), but current consensus holds that edge effects typically extend 150 meters into a fragment (Bierregaard et al., 1992; Murcia, 1995).

Fragmentation stands out among the ecological challenges that affect protected areas. Knowledge regarding the factors that influence diversity in habitat fragments and their effects on native populations can indicate appropriate strategies and control mechanisms for the management of these areas.

Species of the genus *Drosophila* are used in many areas of biological inquiry as model organisms. These flies are potential candidates for monitoring the degree of environmental disturbance in a given area (Parsons, 1991), as permanent changes in the *Drosophila* life cycle (Prince fauna imply significant biotic changes in the plant, fungus and parasitic wasp species that are associated with different stages of the *Drosophila*^1976^; Chabora et al. 1979). Changes in temperature and humidity are known to affect vital parameters in *Drosophila* species, including their survival, fertility, development time and other factors that influence population growth rates and viability (Sene et al. 1980; Tidon-Sklorz and Sene 1992; Balanya et al. 2006; Torres and Madi-Ravazzi 2006).

The potential for these flies to serve as environmental indicators is demonstrated by the cosmopolitan character of the group, the sensitivity of the flies to environmental variables and the simplicity of collecting them (Parsons 1991; Foote and Carson 2004).

The drosophilids include many exotic species, comprising a number of species with a long history of invasion. These flies are primarily found in environments disturbed by man, in open areas, or in degraded and urbanized environments which are characterized by a pronounced degree of environmental stress; *D. simulans*, *D. malerkotliana*, *D. melanogaster*, *Scaptodrosophila latifasciaeformis* and *Zaprionus indianus* occupy such environments. However, some native neotropical species, such as *D. willistoni*, occur only in forested areas and protected environments. These species may therefore be used as bioindicators of environmental conditions (Saavedra et al. 1995; Amaral 2004; Ferreira and Tidon 2005; Torres and Madi-Ravazzi 2006; Penariol 2007; De Toni et al. 2007; Schmitz et al. 2007; Gottschalk et al. 2007; Mata et al. 2008, Acurio et al. 2010).

The effects of habitat fragmentation have been studied in numerous taxa, including plants (Bierregaard et al. 1992; Laurance et al. 1998; Oliveira-Filho et al. 2004), birds (Kroodsma, 1984), and invertebrates (Mcgeoch and Gaston 2000; Demite and Feres 2005; Oliveira-Alves et al. 2005). However, few studies (Martins 1989; Amaral 2004; Penariol 2007) have evaluated the effects of edges on the drosophilid fauna, which therefore represents a relatively novel approach.

The semideciduous forest ecosystem within the Atlantic Forest extends along the central and southeast regions of Brazil’s interior. This vegetation type has experienced severe devastation. In the northwestern region of São Paulo, it is now limited to 9% of its original area. Few investigations have focused on understanding and protecting the species biodiversity associated with the semideciduous forest (SMA/IF 2005; Kronka et al. 1993) To contribute to the knowledge of the fauna of this region and to establish conservation and monitoring strategies for Atlantic Forest fragments, this study evaluated ecological parameters of Drosophilidae as well as the use of these organisms in studies addressing size and edge effects in one of the last remaining fragments of semideciduous forest in São Paulo State, Brazil.

## Materials and methods

### Study area and collection methods

**Figure 1:**
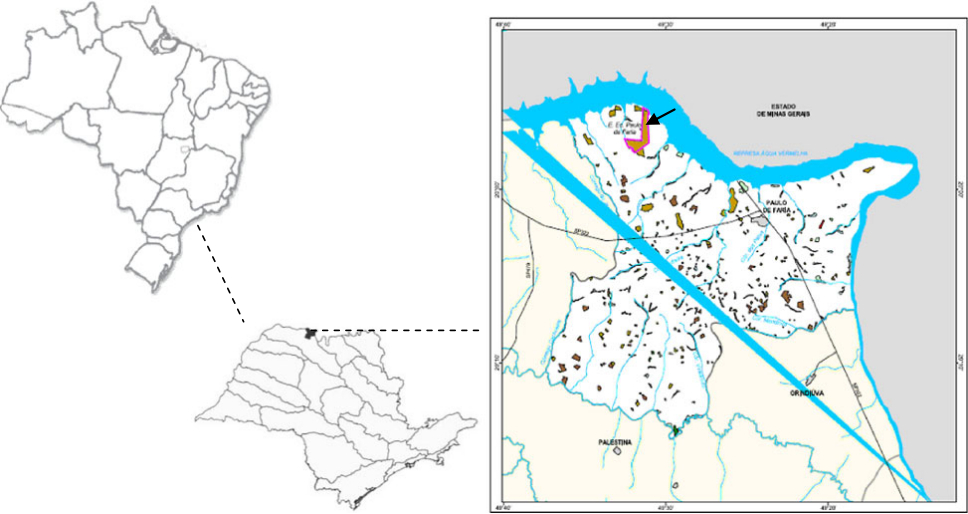
**Geographic location of Paulo de Faria, in northern region of the state of São Paulo-Brazil and location of Estação Ecológica de Paulo de Faria.**

Flies were collected at the Ecological Station of Paulo de Faria (19° 55’ to 19° 58’ S and 49° 31’ to 49° 32’ W) in São Paulo State, Brazil, which is a 435-hectare fragment of seasonal, semideciduous forest (Figure 1). The historic vegetation of this region was a mesophytic semideciduous forest, which was altered for use as pastures and for various monocultures; during the sample period, these crops included corn and cane sugar. This region is characterized by a well-pronounced dry season that accounts for less than 15% of annual precipitation (Barcha and Arid, 1971and unpredictable rainfall at the beginning of the rainy season (Rossa-Feres and Jim, 2001). The average annual temperature and precipitation are 27°C and 127.67 mm, respectively.

**Figure 2:**
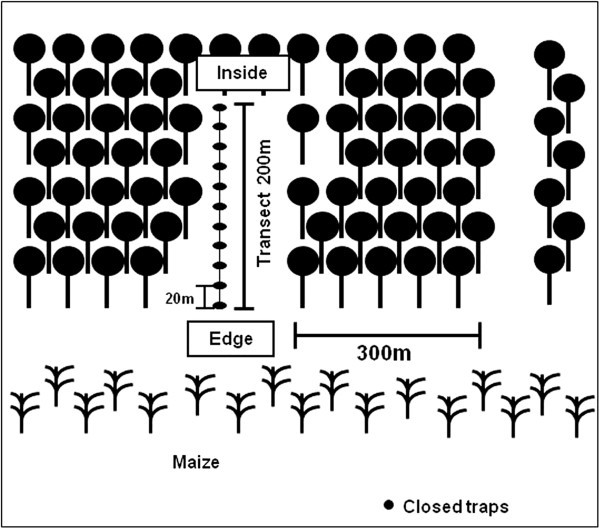
**Drawing the sample used for collection in the fragment.**

Collections were performed monthly, from September 2004 to August 2005, along a 200-meter transect (Figure 2) extending from the edge toward the interior of the fragment. Eleven collection points were distributed along the transect at 20-meter intervals (at 0, 20, 40, 60, 80, 100, 120, 140, 160, 180 and 200 meters). To catch flies, closed traps (Penariol et al., 2008) were placed approximately 1.5 m above the soil surface. The traps contained bait prepared with macerated banana and biological yeast (*Saccharomyces cerevisiae*) and were left at the collection sites for a period of three days.

The flies captured in the closed traps were transferred directly to glass tubes. Subsequently, the specimens were transferred to bottles containing standard culture medium and transported to a laboratory. Specimens were identified according to a standard process using identification keys and, when necessary, by examining the aedeagus (Freire-Maia and Pavan 1949; Kaneshiro 1969; Vilela 1983).

### Statistical analysis

The efficiency of the sampling methodology was evaluated using richness accumulation curves and via richness estimates obtained with the Abundance Coverage Estimator (ACE) and Incidence Coverage Estimator (ICE) provided in the Estimate Swin 7.0 program (Colwell, 2004).Species abundance and richness were analyzed descriptively from graphics prepared in Microsoft Excel 7.0 for Windows. Comparisons between the species distribution and distance along the transect were performed via Analysis of Dependence (Anadep, Cordeiro, 1987).

## Results and discussion

**Figure 3:**
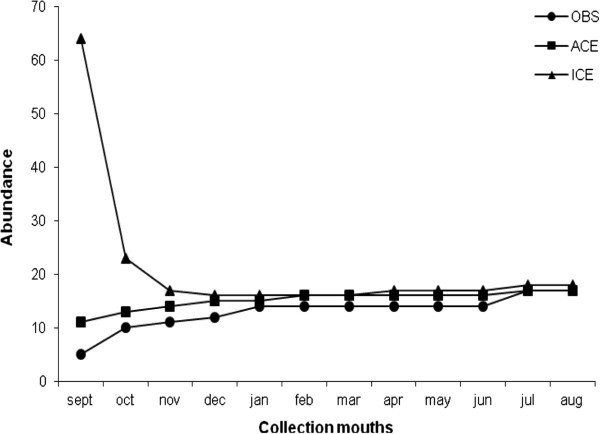
**Curves of accumulation of richness estimated by methods ACE and ICE compared with the richness observed (OBS).**

A total of 6,832 drosophilids distributed among 17 species were captured along the transect, and curves to estimate richness were calculated using the ACE and ICE methods. Both richness estimators exhibited a trend toward stabilization, which demonstrates efficient sampling (Figure 3). Four distinct patterns of species abundance emerged (Table 1). The abundance of the species *D. simulans*, *D. malerkotliana* and Z. *indianus* was high at sampling points near the edge and decreased toward the interior of the fragment. The opposite pattern was observed for *D. willistoni*, which exhibited a high abundance within the fragment and became less common toward the edge. The species S. *latifasciaeformis* was observed only at the fragment edge (up to 60 meters from the edge), while *D. mediostriata* was collected only within the fragment (inward of 120 meters). Other species exhibited no patterns in their abundance related to the distance from the fragment edge.

**Table 1:** **Abundance of drosophilid species collected at each point of transect across the period of collection**

Group	Species	Edge distance in meters											Total
		0	20	40	60	80	100	120	140	160	180	200	
*melanogaster*	*D. simulans*	503	317	228	232	145	139	109	137	84	56	69	2.019
	*D. malerkotliana*	33	24	31	20	6	9	4	0	3	0	2	132
*willistoni*	*D. willistoni*	43	41	76	127	213	233	224	304	291	225	320	2.097
	*D. nebulosa*	28	11	8	12	11	11	7	11	9	19	16	143
*cardini*	*D. polymorpha*	48	29	36	26	37	8	12	26	39	26	29	316
*guarani*	*D. ornatifrons*	0	7	12	1	7	3	5	7	10	7	15	74
*tripunctata*	*D. mediopunctata*	0	7	29	0	0	0	1	9	4	2	3	55
	*D. mediostriata*	0	0	0	0	0	0	3	3	1		2	9
*annulimana*	*D. ararama*	7	4	2	5	0	4	3	2	6	3	8	44
*immigrans*	*D. immigrans*	0	0	0	1	0	1	0	0	0	1	2	5
*saltans*	*D. sturtevanti*	74	67	57	74	70	78	67	105	84	89	101	866
	*D. prosaltans*	8	5	7	9	7	1	9	7	20	15	8	96
	*D. austrosaltans*			3	1	11	4	7	6	6	3	5	46
*repleta*	*D. mercatorum*	27	32	34	19	22	24	25	22	29	14	20	268
	*D. paranaensis*	21	50	38	19	17	14	16	18	12	19	27	251
Others drosophilids	*Z. indianus*	135	91	60	37	25	2	7		1	1		359
	*S. latifasciaeformis*	30	10	9	3								52
Species richness		12	14	15	15	12	14	15	13	15	14	15	6.832

**Figure 4:**
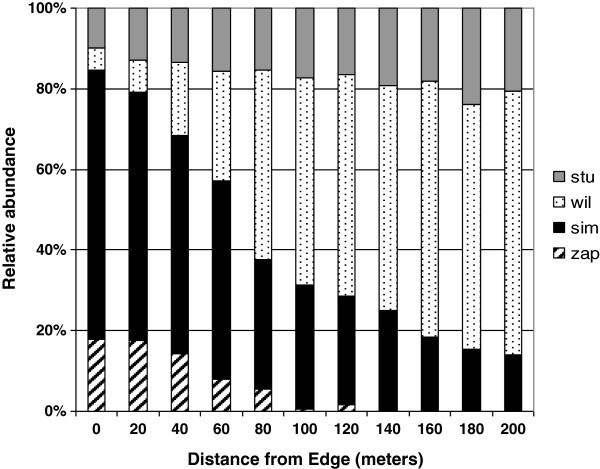
**Relative abundance of species collected in the transect (edge-interior of the wood).***D. sturtevanti* (stu), *D. willistoni* (wil), *D. simulans* (sim) and *Zaprionus indianus* (zap).

*Drosophila simulans* was the dominant species up to 60 meters along the transect, with 63% of its total abundance being recorded within this edge region. *D. willistoni* dominated the collections beyond 80 meters, where 86% of its total abundance was found. Z. *indianus* was the second most abundant species in the edge region up to 20 meters, with 63% of its total abundance being concentrated here, and this species occurred up to 80 meters from the fragment edge. The species *D. sturtevanti*, which was one of the most abundant species, was collected along the entire length of the transect (Figure 4).

**Figure 5:**
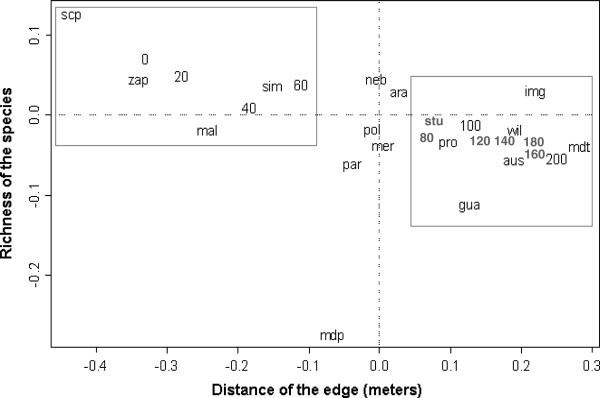
**Analysis of dependence (ANADEP), relating the distance of the edge and richness of the species.** The points of 0 the 60 correspond to the edge area and of 80 the 200 to the inside of the fragment. *S. latifasciaeformis* (scp), *Z. indianus* (zap), *D. malerkotliana* (mal), *D. simulans* (sim), *D. mediopunctata* (mdp), *D. paranaensis* (par), *D. nebulosa* (neb), *D. polymorpha* (pol), *D. mercatorum* (mer), *D. ararama* (ara), *D. sturtevanti* (stu), *D. prosaltans* (pro), *D. guarani* (gua), *D. willistoni* (wil), *D. austrosaltans* (aus), *D. immigrans* (img), *D*. *medioatriata* (mdt).

The Anadep data, shown in Figure 5, indicate an association between the distance from the edge and species richness. According to this analysis, the species abundance data along the transect formed two groups, the first of which extended from 0 to 60 meters (edge region) and the other from 80 to 200 meters (interior region). The species *S. latifasciaeformis*, *Z. indianus*, *D. malerkotliana* and *D. simulans* were more closely associated with the edge of the fragment, while *D. mediostriata*, *D. immigrans*, *D. austrosaltans*, *D. willistoni* and *D. ornatifrons* were associated with the fragment’s interior. The statistical analysis confirmed the descriptive trends obtained from charts, which suggested a higher abundance of invasive species in the edge region (up to 60 meters) and of neotropical species in the interior (beyond 60 meters from the edge).

These results indicate that the effects of the edge on the drosophilid assemblage in this fragment extended to 60 meters. The edge-interior transition was demonstrated by a shift from dominance of *D. simulans* to dominance of *D. willistoni*. Scientific consensus maintains that these effects can extend up to 500 meters from the edge, but an edge-effect distance of 100 meters has been found to be typical for diverse flora and fauna (Laurence, 2000).

In this study, the edge favored the dominance of invasive species, such as Z. indianus and D. simulans, and limited the occurrence of native species, such as D. willistoni. In a study conducted in different areas of the Cerrado, (Mata et al. 2008) observed that neotropical drosophilid species were more abundant in undisturbed forests, while exotic and generalist species were dominant in disturbed forests, grasslands and urban areas. These authors identified five species as indicators of undisturbed forest: D. willistoni, D.ornatifrons, D. mediopunctata, D. maculifrons and D. paraguayensis. Of these species, D. willistoni, D. ornatifrons, D. mediopunctata were also collected in the present study, in the fragment interior.

*Z. indianus* is a species that has only recently been introduced in Brazil. On its continent of origin (Africa), this species occupies open savanna (Vilela, 1999). The species’ native habitat may partly explain its high abundance only at sampling points that were very near the edge. *Z. indianus* is one of the most successful colonizing species in the genus (Chassagnard and Tsaca, 1993), as it utilizes multiple food sources and displays plasticity with respect to climate (Parkash and Yadav, 1993).

*D. simulans* is also an exotic species, as are many members of the neotropical drosophilid fauna. This species has been associated with open and/or urban habitats and is resistant to low humidity conditions (Saavedra et al. 1995; Amaral 2004; Ferreira and Tidon, 2005; Torres and Madi-Ravazzi 2006; Schmitz et al. 2007; Mata et al. 2008), which may explain its high dominance at the edge. This species also occurs in the fragment’s interior, though this occurs primarily during the dry season (Penariol, 2007).

*Drosophila willistoni*, a native neotropical species, was dominant in the fragment’s interior. Data from the literature confirm that this species occurs mainly in forested areas (Saavedra et al. 1995; Amaral 2004; Torres and Madi-Ravazzi 2006). (Ferreira and Tidon 2005) also observed that the species of the family Drosophilidae endemic to the Cerrado biome were unable to invade the city of Brasília (an urban environment associated with varying degrees of habitat stress).

Studies on other organisms in the region of the Ecological Station of Paulo de Faria reinforce the need for monitoring this area to preserve its biodiversity. (Gomes and Noll 2009) compared the diversity and richness of social wasps among three fragments of semideciduous forest located in Paulo de Faria, Pindorama and Neves Paulista. The vegetation in these areas is in different stages of regeneration, and these investigators found that the wasp community of Paulo de Faria showed the lowest species diversity and the greatest abundance. According to these authors, this pattern can be explained by the absence of ecological corridors that limit dispersal.

The results of the present study revealed a distribution gradient in the abundance of drosophilid species along an edge-interior transect in a forest fragment. The study established an edge extent of 60 meters. Moreover, the edge region was characterized by the presence of the invader species *D. simulans* and *Z. indianus*, while the native species *D. willistoni* was associated with the forest interior. These findings indicate that the method presented here is efficient for evaluating edge effects and their extent and can be used in the development of management strategies that aim to preserve forest fragments and detect ecosystem disturbance.
